# Effects of Large Deformation and Velocity Impacts on the Mechanical Behavior of Filled Rubber: Microstructure-Based Constitutive Modeling and Mechanical Testing

**DOI:** 10.3390/polym12102322

**Published:** 2020-10-11

**Authors:** Wei Wei, Yong Yuan, Xiaoyu Gao

**Affiliations:** School of Civil and Hydraulic Engineering, Huazhong University of Science and Technology, Wuhan 430074, China; 2018210230@hust.edu.cn (Y.Y.); m201273050@hust.edu.cn (X.G.)

**Keywords:** filled rubber, constitutive modeling, hyper-viscoelastic, large deformation, velocity impact, mechanical testing

## Abstract

Filled rubber has been extensively used in the repairing, retrofitting, and protecting of civil infrastructures due to its superior physical and mechanical properties. However, effects of large deformation and velocity impacts on the mechanical behavior of filled rubber are not well recognized, one of the major challenges in the past investigations is that the material exhibits significant nonlinearity and sensitivity to velocity. This paper presents a hyper-viscoelastic constitutive modeling and experimental study to capture both the hyperelastic and viscoelastic behaviors of filled rubber under large shear deformation and velocity impacts. Motivated by the micro-mechanism of filled rubber, the constitutive modeling consists of an equilibrium element in parallel with an improved Maxwell element to incorporate both nonlinear hyperelasticity and rate-dependent performance governed by the readjustment and rearrangement of molecular chains in the material. A new strain energy function is developed and the physical description of parameters in the strain energy function is highlighted. The Clausius-Duhem inequality is employed to consider the thermodynamic consistency of the model. Then, stress relaxation property and stress-strain response of filled rubber upon cyclic shear loading with different strain rates (ranging from 0.08 to 12.0 s^−1^) are experimentally studied, and some key observations are summarized. Subsequently, a “Gau-Poly” function is proposed based on the experimental data to describe the viscoelastic property of filled rubber versus strain and strain rate. Finally, stress-strain relationship and hysteretic area obtained from the experimental results were compared with the numerical results of the model, good agreement was achieved and the capacity of the model to accurately reproduce the mechanical behavior of filled rubber under a wide range of deformation and velocity impacts was verified.

## 1. Introduction

**Figure 1:**
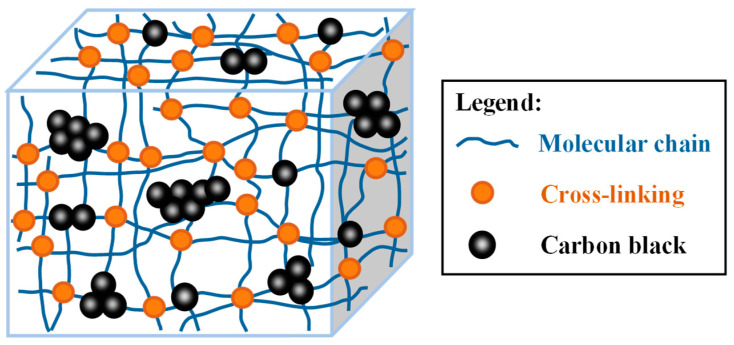
Illustration of microstructure of a carbon black filled rubber.

Filled rubber is an important class of materials due to its favorable flexibility, energy dissipation, self-centering, and other properties [1,2]. It has been extensively employed in isolation bearings, expansion joints, shock absorbers, and other civil engineering applications as a reliable method to mitigate the effects of vibration and dynamic impacts on structures, such as highway bridges and buildings [3,4,5]. The majority of these materials are filled with active fillers, such as carbon black, to remarkably enhance the properties, including stiffness, strength, damping, and abrasion resistance [6,7]. An illustration of the microstructure of a carbon black filled rubber is presented in Figure 1, it shows that the material comprises a rubber matrix, fillers, and rubber-filler interface. The carbon black particles are randomly distributed in the rubber matrix and are clustered into irregularly shaped aggregates with varying sizes and spacing. Moreover, these components further consist of three-dimensional networks, which include a large number of randomly oriented molecular chains with a broad range of lengths that are often cross-linked at junctions or become entangled among themselves [8,9].

Filled rubber-based structural devices are easily exposed to blast, earthquake, and other dynamic impacts ranging from low to high strain rates [10]. Previous experimental studies have revealed that the filled rubber has a complex nonlinear behavior under large deformation and high velocity impacts [11]. The stress-strain response of filled rubber is highly dependent on deformation rate and exhibits significant hysteresis upon cyclic shear loading, resulting in a series of undesired design problems in engineering practices [12]. Although the bilinear hysteretic model is a widely employed modeling approach, it excludes many of the advanced mechanical behaviors of filled rubber, such as strong nonlinear and hardening properties, thus it would yield inaccurate design results [13]. Therefore, the development of a high fidelity constitutive model of filled rubber under a wide range of strains and strains rates is imperative and contributes to the numerical simulation during the design process.

Currently, several sophisticated models and numerical computation are available in the literature to address different aspects of mechanical properties of filled rubber—e.g., Lion [14], Jankowski [15], Li et al. [16], Gjorgjiev et al. [17], Sahu et al. [18], Khajehsaeid et al. [19], and more recently Mokhireva et al. [20]. However, most available researches focused on the uniaxial tension or compression tests of the material, whereas limited experimental works regarding simple shear deformation have been reported, which essentially play an important role in analyzing the mechanical behavior of rubber-based structural devices under seismic excitation in civil engineering. It was found that the reasons are twofold: firstly, research interest in the shear deformation has merely grown in recent years. Secondly, additional measurement difficulty is encountered when filled rubber is under more challenging conditions like large deformation and high velocity impacts [21]. More importantly, those models have a drawback in that their estimations are unsatisfactory under large strains and high velocity circumstances. The viscosity effect of the material is roughly expressed via a Prony series approach [22], yet the nature and physical basis of the behavior of filled rubber is not well recognized.

On the other hand, there is another approach to develop the constitutive model based on the characteristics of microstructure of filled rubber and micro-mechanics owing to the fact that its mechanical behavior is mainly governed by the different cross-linking networks in the material [23]. Tomita et al. investigated the monotonic and cyclic behavior of carbon black filled rubber through a molecular-chain network model, but the time-dependent nature of the material is yet to be addressed [24]. Bergström et al. studied the microstructure of carbon black filled chloroprene rubber and concluded that the mechanical response of filled rubber incorporates equilibrium and rate-dependent components [25]. Pouriayevali et al. investigated the relationship between the readjustment of the molecular chain in rubber and its stress relaxation property in the macro level. Based on this, they developed a relaxation time model to describe the strain rate sensitivity of rubber material [26].

Following the argument of Bergström et al. and inspired by the micro-mechanical behavior of filled rubber, the main objective of this study is to propose a constitutive model to capture both hyperelastic and viscoelastic properties of filled rubber under extensive ranges of strains and strain rates, especially attempting to consider the influence of fillers on altering the behavior of the material and to develop the viscosity law in a thermodynamically consistent way. The first part of the model comprised of a hyperelastic equation based on a newly developed strain energy function to represent the rate-independent response. The other part of the model incorporated an improved Maxwell element to consider the nonlinearity and rate-dependent response of filled rubber, multiplicative kinematics decomposition of deformation gradient tensor into elastic and inelastic parts was conducted to correlate the overstress tensor with strain rate. Besides, the Clausius-Duhem inequality was employed to ensure the thermodynamic consistency [27], the Helmholz free energy was separately stored in the above-mentioned two parts of the model, and an associated nonlinear viscosity coefficient was derived. Subsequently, a strain-controlled mechanical testing program covering large deformation (200% shear strain) and various velocity impacts (strain rate ranging from 0.08 to 12.0 s^−1^) was performed at room temperature to investigate the stress-strain relationship of the material. Then, hyperelastic parameters were identified straightforward based on the testing results and a “Gau-Poly” function was proposed to describe the viscoelastic property of filled rubber versus strain and strain rate. Finally, numerical results from the model were compared with experimental data.

## 2. Constitutive Modeling

**Figure 2:**
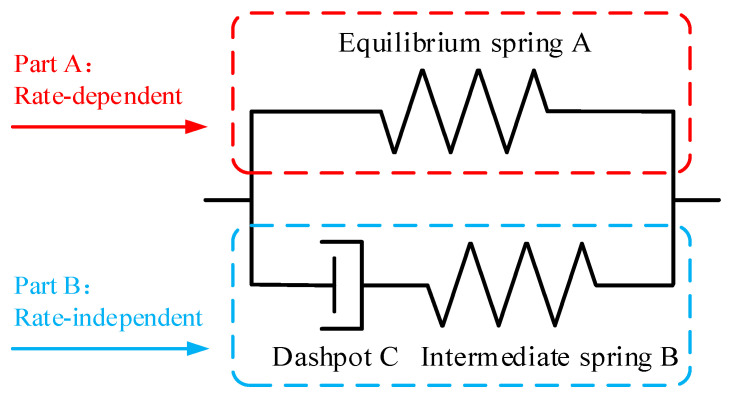
Illustration of the hyper-viscoelastic model.

Motivated by the micro-mechanism of filled rubber, a hyper-viscoelastic model is proposed to incorporate its hyperelastic and viscoelastic characteristics. The model is decomposable in two parallel parts, as depicted in Figure 2—part A is modeled by an equilibrium spring A to represent the rate-independent equilibrium response, while part B is consists of a generalized Maxwell element to describe the rate-dependent instantaneous response. It is an improvement on the traditional Maxwell element to incorporate nonlinearity in both spring and dashpot elements, contributing to model the rapid readjustments of short chains as well as the sluggish rearrangement and excessive entanglement of long chains in the material.

When loading rate is very slow, no stress is transferred through the dashpot, and the equilibrium spring A is the only source of stress tensor. Conversely, when an infinitely high rate is applied, the dashpot C does not have enough time to complete the relaxation processes—all the deformation is undergone by the intermediate spring B. These two boundary states, respectively, correspond to equilibrium and instantaneous responses of filled rubber, which can be described by hyperelastic equations based on the strain energy function. Between the limiting states, the behavior of the generalized Maxwell element is rate-dependent and the stress-strain behavior of the material is governed by a hyper-viscoelastic constitutive law.

### 2.1. Hyperelasticity

**Figure 3:**
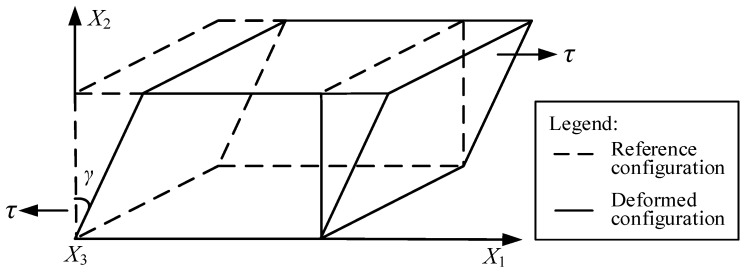
Reference and deformed configurations of simple shear deformation.

Considering a particle located at position X in a material, when simple shear deformation is applied on the body, as illustrated in Figure 3, new position *x* of the particle in the deformed configuration can be described by the reference configuration as:

(1) $$\left\{ \begin{array}{l} x_{1}=X_{1}+\gamma X_{2}\\ x_{2}=X_{2}\\ x_{3}=X_{3} \end{array}\right.$$

where *γ* is the amount of shear strain along the *X*_1_ direction.

Using Equation (1), total deformation gradient tensor **F** in the finite strain kinematic framework is defined as:

(2) $$F=\nabla x=\left[\begin{array}{ccc} 1 & \gamma & 0\\ 0 & 1 & 0\\ 0 & 0 & 1 \end{array}\right]$$

Further, the left Cauchy-Green deformation tensor **B** is obtained by:

(3) $$B=FF^{T}=\left[\begin{array}{ccc} 1+\gamma^{2} & \gamma & 0\\ \gamma & 1 & 0\\ 0 & 0 & 1 \end{array}\right]$$

where **F**^T^ is the transposed matrix of **F**.

If Equation (3) holds, the first, second, and third strain invariants of **B** are:

(4) $$\begin{array}{l} I_{1}^{}=\mathrm{tr}Β=3+\gamma^{2}\\ I_{2}^{}=\frac{1}{2}\left[{\left(\mathrm{tr}Β\right)}^{2}-\left(\mathrm{tr}{Β}^{2}\right)\right]=3+\gamma^{2}\\ I_{3}^{}=\det Β=1 \end{array}$$

where tr(·) and det(·) are trace and determinant operators, respectively.

The bulk modulus of filled rubber is much greater than its shear modulus, suggesting that a hypothesis of Poisson’s ratio *ν* = 0.5 is reasonable. Thus, applying isotropy and incompressibility yields:

(5) $$\det F=\det\;F_{A}=\det\;F_{B}=1$$

(6) $$S\equiv (\det F)T=-p1+S_{E}$$

where **S** and **T** are weighted Cauchy and Cauchy stress tensors, respectively. Subscript *E* represents the extra part of **S**. *p* is the hydrostatic pressure of **S**; **1** is a second-order identity tensor.

Following the framework of hyper-viscoelastic model, **S***_E_* at any time is the sum of equilibrium stress tensor $S_{E}^{eq}$ and over stress tensor **$S_{E}^{ov}$**:

(7) $$S_{E}=S_{E}^{eq}+S_{E}^{ov}$$

The stress tensor is determined by the differentiation of strain energy function (SEF) with respect to **B**:

(8) $$S_{E}^{eq}=2\frac{\partial W^{eq}}{\partial I_{1A}}B_{A}-2\frac{\partial W^{eq}}{\partial I_{2A}}B_{A}^{-1}$$

(9) $$S_{E}^{ov}=2\frac{\partial W^{ov}}{\partial I_{1B}}B_{B}-2\frac{\partial W^{ov}}{\partial I_{2B}}B_{B}^{-1}$$

where *W^eq^* and *W^ov^* represent the SEFs of equilibrium spring A and intermediate spring B, respectively. *I*_1*A*_ and *I*_2*A*_ denote first and second invariants of **B***_A_* in the equilibrium spring, respectively. *I*_1*B*_ and *I*_2*B*_ are that of **B***_B_* in the intermediate spring, respectively.

Since an appropriate SEF plays a crucial role in describing the mechanical behavior of filled rubber, a large number of SEFs are developed and frequently utilized in the literature, such as the Mooney-Rivlin model, Neo-Hookean model, Ogden model, and Yeoh model [28,29]. Although the abovementioned models provide acceptable agreement with the experimental results in extension and compression modes, several drawbacks of these models are revealed, especially the fact that they fail to accurately reproduce the highly nonlinear stress-strain response of filled rubber under large shear deformation and high velocity impacts [30]. Therefore, a new SEF should be developed based on the mechanical characteristic of filled rubber under considered conditions in the study.

Generally, the SEF is expressed as the sum of two functions associated with *I*_1_ and *I*_2_ [31,32],

(10) $$W(I_{1},I_{2})=\int f(I_{1})\;dI_{1}+\int g(I_{2})\;dI_{2}$$

where

(11) $$f(I_{1})=\frac{\partial W}{\partial I_{1}};\;g(I_{2})=\frac{\partial W}{\partial I_{2}}$$

In order to ensure the proposed SEF is capable of representing the basic situations of filled rubber, it must satisfy the following requirements. Firstly, the SEF should vanish for the undeformed configuration of filled rubber.

(12) $$W_{\left(I_{1}=3\right)}=0$$

Secondly, the SEF and obtained stress tensors approached infinity when filled rubber is subjected to infinite deformation.

(13) $$\underset{\gamma \to +\infty }{\lim}W=+\infty$$

(14) $$\underset{\gamma \to +\infty }{\lim}\frac{\partial W}{\partial I_{1}}=+\infty$$

Moreover, a good SEF should have a simple mathematical structure with minimal number of parameters to describe the essential features of the material and to ensure the easy application. Based on these considerations, a five-constant polynomial SEF is proposed in Equations (15) and (16) to consider the “S” shaped strong nonlinear behavior of filled rubber associated with the complex entropic rearrangement of molecular chains in the material.

(15) $$\begin{array}{l} W^{eq}=C_{1}{}^{eq}(I_{1A}-3)+\frac{2}{3}C_{2}{}^{eq}{\left(I_{1A}-3\right)}^{\frac{3}{2}}+\frac{1}{2}C_{3}{}^{eq}{\left(I_{1A}-3\right)}^{2}+\frac{2}{5}C_{4}{}^{eq}{\left(I_{1A}-3\right)}^{\frac{5}{2}}\\ +\frac{C_{5}{}^{eq}}{3}{\left(I_{1A}-3\right)}^{3} \end{array}$$

(16) $$\begin{array}{l} W^{ov}=C_{1}{}^{ov}(I_{1B}-3)+\frac{2}{3}C_{2}{}^{ov}{\left(I_{1B}-3\right)}^{\frac{3}{2}}+\frac{1}{2}C_{3}{}^{ov}{\left(I_{1B}-3\right)}^{2}+\frac{2}{5}C_{4}{}^{ov}{\left(I_{1B}-3\right)}^{\frac{5}{2}}\\ +\frac{C_{5}{}^{ov}}{3}{\left(I_{1B}-3\right)}^{3} \end{array}$$

where $C_{i}^{eq}$ (*i* = 1~5) are responsible for the material parameters of equilibrium stress, while $C_{i}^{ov}$ (*i* = 1~5) are those of overstress.

It is seen that the proposed SEF is also a particular case of the well-known Rivlin’s expression:

(17) $$W_{\left(\mathrm{Rivlin}\right)}=\sum_{i=0,j=0}^{\infty }C_{ij}{\left(I_{1}-3\right)}^{i}{\left(I_{2}-3\right)}^{j}$$

When small deformation like initial linear range is applied on filled rubber, the SEF is simplified as:

(18) $$W_{\left(\mathrm{Small}\;\mathrm{deformation}\right)}=C_{1}(I_{1}-3)$$

Noting that the Neo-Hookean model is calculated by the material chain density *n*, Boltzman’s coefficient *k*, and absolute temperature *T* [33], which is shown in Equation (19)

(19) $$W_{\left(\mathrm{Neo}-\mathrm{Hookean}\right)}=\frac{1}{2}nkT(I_{1}-3)$$

The Neo-Hookean model is based on the statistical thermodynamics of molecular chains in the material, proceeding to compare Equation (18) and Equation (19) leads to:

(20) $$C_{1}=\frac{1}{2}nkT$$

therefore, the physical meaning of parameters in the SEF is highlighted, *C*_1_ is related to parameters of *n*, *k*, and *T*.

On the other hand, the behavior of filled rubber under large deformation is mainly dominated by the parameters *C*_4_ and *C*_5_ in the proposed SEF. From the perspective of the microstructure of the material, large deformation involves a greater proportion of molecular chains and is determined by their limiting extensibility.

In addition, it should be noted that ignoring *I*_2_ in the proposed SEF may induce some but not significant errors in describing the advanced aspect like poynting-type effect in rubber material [34], it suffices to illustrate the core concept in the study.

Substituting Equation (15) into Equation (8), the hyperelastic relation for the shear component of equilibrium stress tensor is given by:

(21) $$\tau_{}^{eq}=2(C_{1}^{eq}\gamma_{A}+C_{2}^{eq}\gamma_{A}^{2}\mathrm{sgn}(\gamma_{A})+C_{3}^{eq}\gamma_{A}^{3}+C_{4}^{eq}\gamma_{A}^{4}\mathrm{sgn}(\gamma_{A})+C_{5}^{eq}\gamma_{A}^{5})$$

(22) $$\mathrm{sgn}(\gamma )=\left\{ \begin{array}{l} +1\;;\;\gamma >0\\ \;0\;;\;\gamma =0\;\\ -1\;;\;\gamma <0 \end{array}\right.$$

where $C_{i}^{eq}$ (*i* = 1~5) are rate-independent material parameters.

Substitution of Equation (16) into Equation (9) yields the shear component of overstress tensor:

(23) $$\tau_{}^{ov}=2(C_{1}^{ov}\gamma_{B}+C_{2}^{ov}\gamma_{ov}^{2}\mathrm{sgn}(\gamma_{B})+C_{3}^{ov}\gamma_{B}^{3}+C_{4}^{ov}\gamma_{B}^{4}\mathrm{sgn}(\gamma_{B})+C_{5}^{ov}\gamma_{B}^{5})$$

where $C_{i}^{ov}$ (*i* = 1~5) are rate-dependent material parameters.

### 2.2. Hyper-Viscoelastic

**Figure 4:**
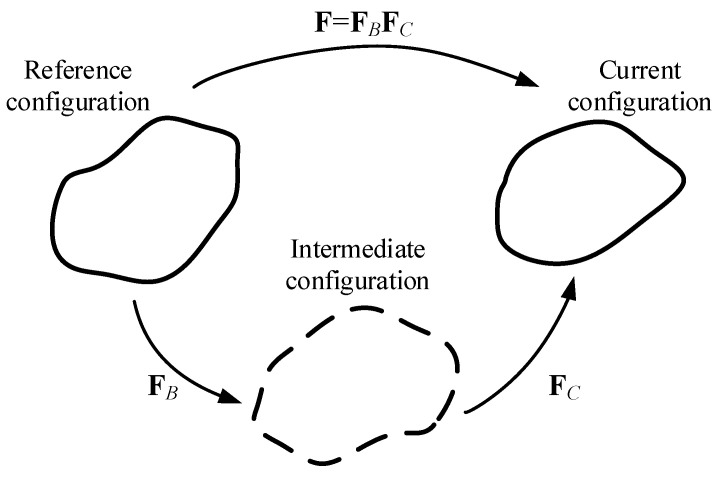
Illustration of multiplicative kinematics decomposition of **F**.

As discussed previously, between the two limiting states (i.e., very slow and fast loading rates), the Maxwell element can be decomposed within the framework of a multiplicative kinematics decomposition of **F** [35], as shown in Equation (24) and Figure 4.

(24) $$F=F_{B}F_{C}$$

where **F***_B_* and **F***_C_* represent the deformation gradient tensors of spring B and dashpot C, respectively.

Following Equation (24), the left Cauchy-Green deformation tensors of intermediate spring B and dashpot C are deduced, respectively.

(25) $$B_{B}=F_{B}^{}F_{B}^{T}$$

(26) $$B_{C}=F_{C}^{}F_{C}^{T}$$

After taking the material time derivative of **B***_B_* and **F***_B_*, we have

(27) $${\dot{B}}_{B}={\dot{F}}_{B}F_{B}^{T}+F_{B}{\dot{F}}_{B}^{T}$$

(28) $${\dot{F}}_{B}=\frac{d(FF_{C}^{-1})}{dt}=LF_{B}-F_{B}L_{C}$$

where **L** is the total velocity gradient tensor and **L***_C_* is the corresponding velocity gradient tensor of dashpot C, which are derived as

(29) $$L=\dot{F}F^{-1}$$

(30) $$L_{C}={\dot{F}}_{C}F_{C}^{-1}=D_{C}+W_{C}$$

where

(31) $$D_{C}=\frac{1}{2}(L_{C}+L_{C}^{T})$$

(32) $$W_{C}=\frac{1}{2}(L_{C}-L_{C}^{T})$$

Substituting Equations (28)–(32) into Equation (27), the following equation is derived.

(33) $${\dot{Β}}_{B}=-2F_{B}^{}D_{C}F_{B}^{T}+B_{B}L^{T}+LB_{B}$$

For an isothermal viscoelastic process, the second law of thermodynamics in the form of the Clausius-Duhem inequality requires that [36]

(34) $$S_{E}\cdot L-\dot{\psi }\geq 0$$

where *ψ* is the Helmholtz free energy.

According to the hyper-viscoelastic model, it is noted that the energy can be only stored in spring A and B, thus, *ψ* has the following form:

(35) $$\psi =W^{eq}(I_{1}{}_{A})+W^{ov}(I_{1B})$$

Taking the material time derivative of *ψ*, the rate of free energy is expressed as:

(36) $$\dot{\psi }=\frac{\partial W^{eq}}{\partial I_{1A}}{\dot{I}}_{1A}+\frac{\partial W^{ov}}{\partial I_{1B}}{\dot{I}}_{1B}$$

considering

(37) $${\dot{I}}_{1A}=1\cdot {\dot{B}}_{A};{\dot{I}}_{1B}=1\cdot {\dot{B}}_{B}$$

thus, Equation (36) can be rewritten as:

(38) $$\dot{\psi }=\frac{\partial W^{eq}}{\partial I_{1A}}1\cdot {\dot{B}}_{A}+\frac{\partial W^{ov}}{\partial I_{1B}}1\cdot {\dot{B}}_{B}$$

On the other hand, according to Equation (24) and Equation (29), the velocity gradient tensor can be decomposed into:

(39) $$\;L=\dot{F}F_{}^{-1}=L_{B}+F_{B}L_{C}F_{B}^{-1}$$

Considering Equation (7), **S***_E_* is the sum of $S_{E}^{eq}$ and $S_{E}^{ov}$, thus

(40) $$S_{E}\cdot L=(S_{E}^{eq}+S_{E}^{ov})\cdot L=S_{E}^{eq}\cdot L+S_{E}^{ov}\cdot L_{B}+(F_{B}S_{E}^{ov}F_{B}^{-1})\cdot D_{C}$$

where

(41) $$S_{E}^{eq}\cdot L\;=\;\mathrm{tr}(S_{E}^{eq}{\dot{B}}_{A}B_{A}^{-1}-S_{E}^{eq}B_{A}L^{T}B_{A}^{-1})=(B_{A}^{-1}S_{E}^{eq})\cdot {\dot{Β}}_{A}-S_{E}^{eq}\cdot L$$

thus

(42) $$S_{E}^{eq}\cdot L=\frac{1}{2}(B_{A}^{-1}S_{E}^{eq})\cdot {\dot{Β}}_{A}$$

By the analogy with Equation (42), the second term on the right side of Equation (40) becomes:

(43) $$S_{E}^{ov}\cdot L_{B}=\frac{1}{2}(B_{B}^{-1}S_{E}^{ov})\cdot {\dot{Β}}_{Β}$$

Substituting Equations (38)–(43) into Equation (34), the inequality function can be transformed into:

(44) $$(\frac{1}{2}(B_{A}^{-1}S_{E}^{eq})-\frac{\partial W^{eq}}{\partial I_{1A}}1)\cdot {\dot{Β}}_{A}+(\frac{1}{2}(B_{B}^{-1}S_{E}^{ov})-\frac{\partial W^{ov}}{\partial I_{1B}}1)\cdot {\dot{Β}}_{Β}+(F_{B}S_{E}^{ov}F_{B}^{-1})\cdot D_{C}\geq 0$$

With the definition of equilibrium stress tensor and overstress tensor expressed in Equations (8) and (9), Equation (44) is reduced to the following equation, implying that the power between overstress tensor and velocity gradient tensor of dashpot C should be non-negative.

(45) $$(F_{B}S_{E}^{ov}F_{B}^{-1})\cdot D_{C}\geq 0$$

For an arbitrary process, the simplest sufficient condition satisfying Equation (45) is:

(46) $$D_{C}=\frac{1}{\eta }F_{B}S_{E}^{ov}F_{B}^{-1};\;\eta >0$$

where *η* is a positive viscosity coefficient.

For the sake of computational simplicity, the validity of Equation (46) is equivalent to the following equation [35]:

(47) $$D_{C}=\frac{1}{\eta }\left\{F_{B}^{T}S_{E}^{ov}F_{B}^{T}{}^{-1}-\frac{1}{3}\left[\mathrm{tr}\left(S_{E}^{ov}\right)\right]1\right\}$$

operating on the inelastic intermediate configuration, we have:

(48) $$F_{B}D_{C}F_{B}^{T}=\frac{1}{\eta }B_{B}{\left(S_{E}^{ov}\right)}^{D}$$

where superscript *D* is the deviatoric component of overstress tensor **$S_{E}^{ov}$**.

Substituting Equation (48) into Equation (33) yields:

(49) $${\dot{B}}_{B}=B_{B}L^{T}+LB_{B}-\frac{2}{\eta }B_{B}{\left(S_{E}^{ov}\right)}^{D}$$

Overall, a thermodynamically consistent hyper-viscoelastic constitutive model is established from a combination of two classes of equations. The first corresponds to the limiting states of the material defined by Equations (21)–(23). The second is linked to the rate-dependent response of the material and associated with part B in the model.

## 3. Mechanical Testing Program

### 3.1. Specimen Preparation and Test Setup

**Figure 5:**
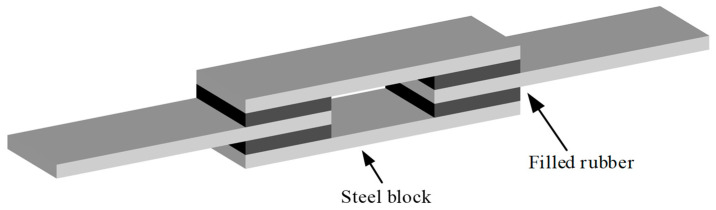
Illustration of the quadruple-lap shear specimen.

Commercially available nitrile rubber was chosen as the base rubber and carbon black N330 was employed as the reinforcing filler to fabricate the specimens. According to the ISO 1827:2007 standard [37], Figure 5 shows the quadruple-lap shear specimen which comprises of four filled rubber layers and rigid steel blocks, the rubber layers are glued to the steel blocks by adhesive material to prevent sliding on the contacting surfaces.

**Figure 6:**
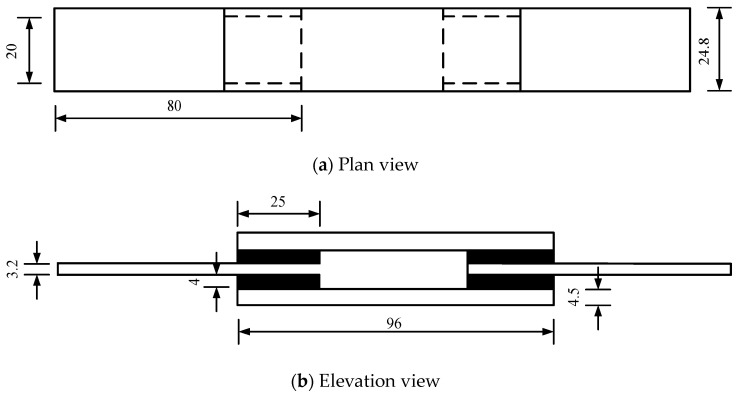
Geometrical sizes of the filled rubber and steel block. (unit: mm).

Figure 6 depicts the geometrical sizes of the filled rubber and steel block in the specimen. Each rubber layer has the following dimensions: 25 mm length, 20 mm width, and 4 mm thickness, the length and width are much greater than the thickness to restrict end rotation of rubber layers during the shear deformation and ensure homogeneous strain under high loading rate condition.

**Figure 7:**
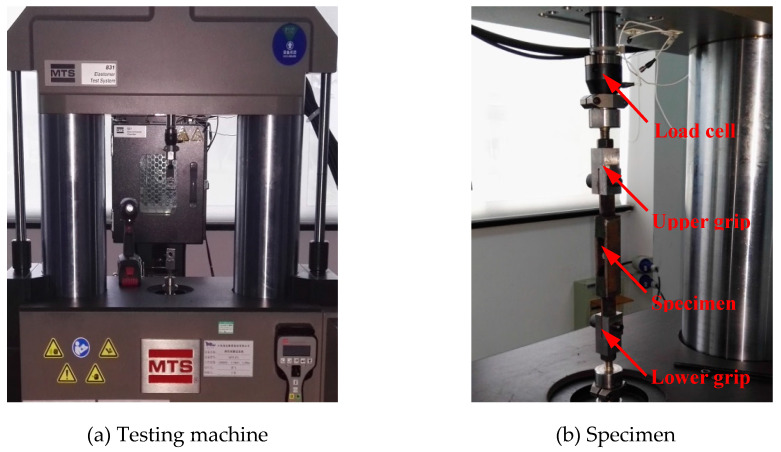
Test setup and specimen.

As presented in Figure 7, a servo-hydraulic MTS 831 elastomer testing machine was employed to provide required loading schemes and measure the corresponding shear force. The quadruple-lap shear specimen was mounted on the apparatus by the upper and lower grips, specified displacement was applied at the top steel block to allow it move freely in the vertical direction with a wide range of strain rates from 0.08 s^−1^ to 12.0 s^−1^. The filled rubber deforms under simple shear deformation while the steel blocks are assumed to be undeformed in the tests. In order to remove the Mullins effect and obtain a relatively stable state of stress-strain curves of the specimens, they were subjected to ten cycles with a maximum shear strain of 200% before the formal tests. All the tests were performed at room temperature.

### 3.2. Stress Relaxation and Cyclic Shear Tests

To assess the stress relaxation behavior of filled rubber, generally speaking, a prescribed step strain is exerted on the specimen and holds for a period ranging from 20–60 min to complete various forms of molecular chain readjustments in the material and reach a relatively stable state. Then, the holding period results in an asymptotical reduction in the stress to approximately one third or less of the stress at the beginning of relaxation, implying that the stress achieves an equilibrium state [38]. Once the holding period was finished, the procedure was repeated by increasing the strain amplitude.

**Figure 8:**
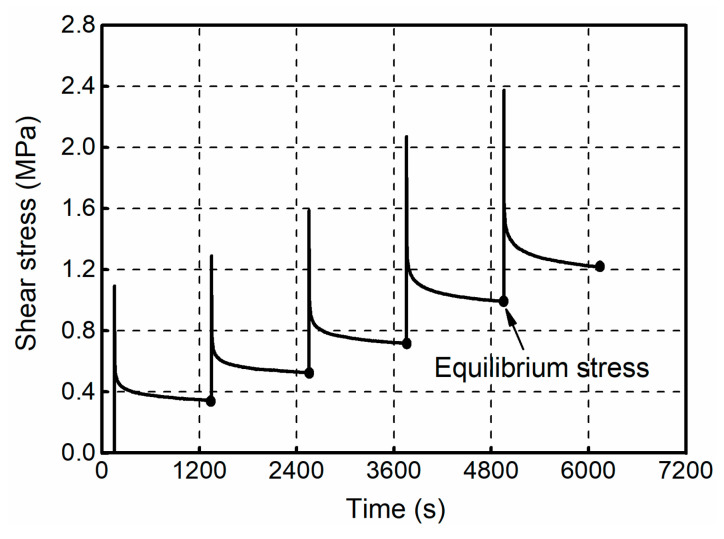
Shear stress obtained from the stress relaxation tests.

In this study, the specimens were successively loaded to strain levels of 25%, 75%, 125%, 175%, and 200%, and held for 1200 s during each step. Figure 8 shows the stress relaxation results of filled rubber, a time shift of 150 s was introduced in the figure to highlight the comparison. It was observed that, during each holding step, the stress undergoes a rapid decrease at the very beginning of the process, almost 50% of the stress relaxation occurs in the first 5 s. Then, the stress gradually converges to a limit value, accompanied by the occurrence of rearrangement of microstructure in the filled rubber. At the end of the holding step, the stress at the termination point was identified as the approximation of equilibrium stress.

**Figure 9:**
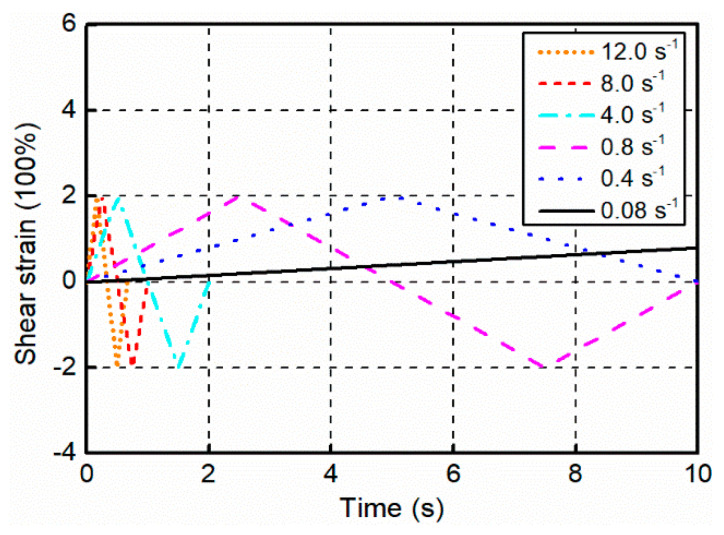
Applied strain histories in the cyclic shear tests.

As shown in Figure 9, cyclic shear tests consist of saw-tooth wave displacement excitation with fully reversed cycles of loading at a maximum strain of 200% to consider general cases of filled rubber-based structural devices used in civil engineering projects. Six different strain rates, including 0.08, 0.4, 0.8, 4.0, 8.0, and 12.0 s^−1^ were employed to investigate the cyclic mechanical performance of filled rubber under various velocity impacts, covering most typical dynamic scenarios that filled rubber undergoes during earthquake excitations. Among the loading rates, the strain rate = 12.0 s^−1^ was used as the approximation and alternative solution of the theoretical instantaneous response to calibrate the material parameters associated with the intermediate spring B owing to the fact that an infinitely fast rate was inaccessible because of the limitation of testing machine.

**Figure 10:**
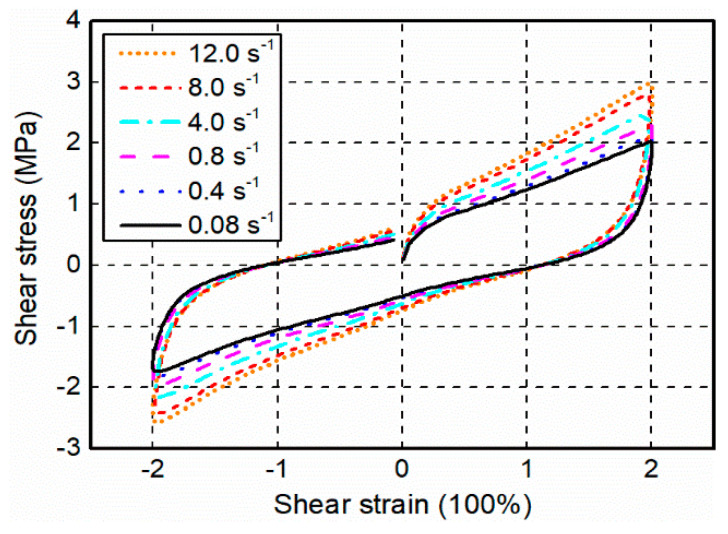
Measured stress-strain responses in the cyclic shear tests.

Figure 10 presents the cyclic shear test results of filled rubber, it is clearly found that the stress-strain responses not only exhibit pronounced nonlinearity with respect to small, moderate, and large strains, but also are very sensitive to the loading rate—specifically, the strain rate dependence is more obvious in the loading process than the unloading process. For any given strain level in the positive loading procedure, the shear stress shows a significant increasing trend when the loading rate increases from 0.08 to 12.0 s^−1^. More specifically, the maximum shear stress in the case of strain rate = 0.08 s^−1^ is 1.94 MPa, while that of strain rate = 12.0 s^−1^ is 2.86 MPa, the corresponding increment of the stress is 47.4%. These unique phenomena can be attributed to the presence of high amount of fillers which alter the microstructure of the material and eventually result in a viscoelastic behavior in the filled rubber.

In addition, geometric dimensions of the specimens were examined after the tests. It was observed that the filled rubber specimens almost retain their original geometric dimensions (i.e., the residual strains are negligible) after the loading tests, indicating the observed phenomenon is amenable to description by the proposed hyper-viscoelastic model.

## 4. Parameter Identification

The identification for the model parameters consists of two procedures, the first is related to the hyperelastic parameters and the second focuses on the viscoelastic parameters.

### 4.1. Hyperelastic Parameters

Parameters of equilibrium stress were directly identified by minimizing the error between the stress relaxation test results and simulated results associated with Equation (21). It should be noted that $C_{5}^{eq}$ was initially taken to be equal to zero because the first four items on the right side of Equation (21) were sufficiently to fit the stress relaxation results of filled rubber. While the parameter calibration for overstress was based on three steps, firstly, the case of strain rate = 12.0 s^−1^ in the cyclic shear tests was selected considering the approximation of infinitely fast rate. Then, the overstress was extracted from the total stress by subtracting the equilibrium stress. Finally, parameters could be estimated in accordance with Equation (23). By applying nonlinear least-squares method, the identified parameters are listed in Table 1.

### 4.2. Viscoelastic Parameters

**Figure 11:**
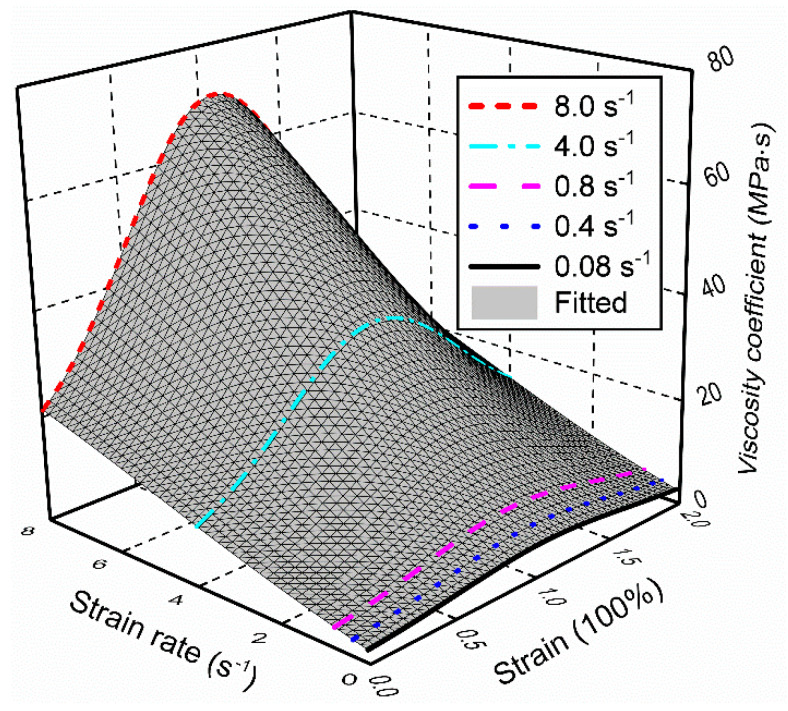
Experimental and fitted results of the viscosity coefficient.

Figure 11 presents the experimental and fitted results of the viscosity coefficient. In particular, the experimental results are first focused—it is easily observed that the viscosity coefficient is positively correlated with the strain rate. For a specified strain level, the viscosity coefficient is increased with the increment of strain rate—this variation is more pronounced in high rate conditions (i.e., strain rate > 0.8 s^−1^). While, for a given strain rate, it is also seen that the viscosity coefficient achieves the maximum value at moderate strain levels, whereas the value is decreased at small and large strains.

Therefore, it is concluded that the viscosity coefficient *η* is closely related to the strain rate *v* and strain *γ* (i.e., *η* = *η* (*γ*, *v*)), this phenomenon is also experimentally demonstrated for styrene butadiene rubber by Fatt et al. [39]. Motivated by the experimental results, a fitting function is introduced to characterize how the viscosity coefficient was affected by the strain and strain rate. In this function, a Gaussian function of *γ* and *v* was implemented in conjunction with a second-order polynomial function of *v* to develop an accurate and robust model for the reproduction of multi-dependent behavior of *η*, which is called “Gau-Poly” function and expressed in the following form:

(50) $$\eta =\{z_{0}+A\exp[-0.5{\left(\frac{\gamma -x_{c}}{w_{1}}\right)}^{2}-0.5{\left(\frac{v-y_{c}}{w_{2}}\right)}^{2}]\}\times (b+cv+dv^{2})$$

where parameters *z*_0_, *A*, *x_c_*, *w*_1_, *y_c_*, and *w*_2_ in the Gaussian function govern the multi-dependence of *η*, while the rest of the parameters *b*, *c*, and *d* are served to describe the strong variation of *η* with strain rate. It is noted that *x_c_*, *w*_1_, and *b* are non-unit parameters.

The parameter identification of viscosity coefficient was done by the following double sub-processes. Firstly, the rate-dependent characteristic of filled rubber was temporarily neglected under a special condition of strain rate = 0.08 s^−1^ and it was reasonable to deem that the *η* was only determined by the Gaussian function in this case [40]. Once the parameters belonging to the Gaussian function were determined, the second step was conducted to identify the rest parameters by approximating the rate-dependent variation in *η*. This arises from the fact that the viscosity coefficient is nonlinearly proportional to the strain rate. The identified parameters and their units are summarized in Table 2—reasonable agreement between the experimental and fitted results is shown in Figure 11, demonstrating that the function has a good generalization ability to predict the variation in viscosity coefficient with varying strains and strain rates.

## 5. Model Application, Validation, and Discussion

In this section, numerical results of the proposed model are compared with the experimental tests to validate how it works in predicting the mechanical behavior of the material.

### 5.1. Monotonic Shear Tests and Model Predictions

**Figure 12:**
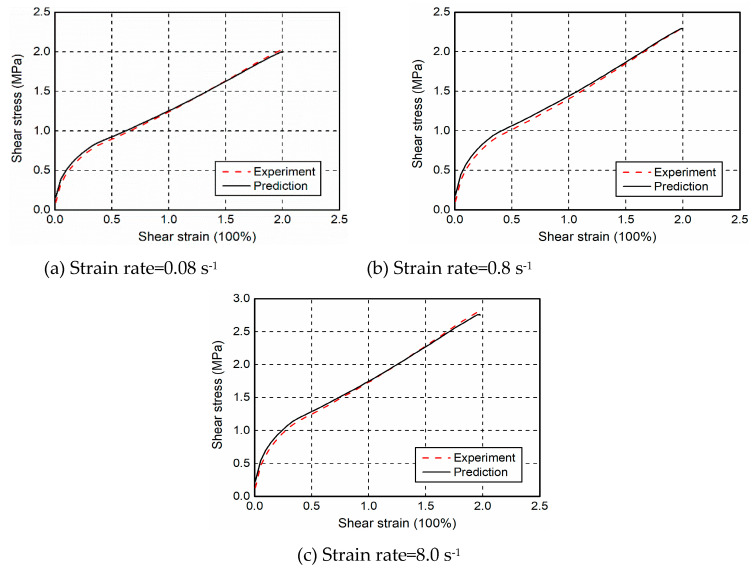
Comparison of monotonic shear tests and model predictions.

To validate the applicability of the proposed constitutive model in predicting the rate-dependent and nonlinear properties of filled rubber, monotonic shear tests with three levels of strain rates, including 0.08, 0.8, and 8.0 s^−1^ were simulated, roughly corresponding to slow, moderate, and high velocity scenarios. By incorporating the proposed model in a MATLAB software code, Figure 12 presents the comparison of experimental results and model predictions. It is seen that the numerical simulations can accurately reproduce the stress-strain response of filled rubber—a good agreement including a rapid stiffness growth at vary small strain amplitudes and rate-dependent performance for different strain rate cases is obtained. Among all the loading schemes, the discrepancy is within the range of 6% and mainly exists at very small strains, resulting from the fact that the fillers substantially increase the initial stiffness of filled rubber.

### 5.2. Cyclic Shear Tests and Model Predictions

**Figure 13:**
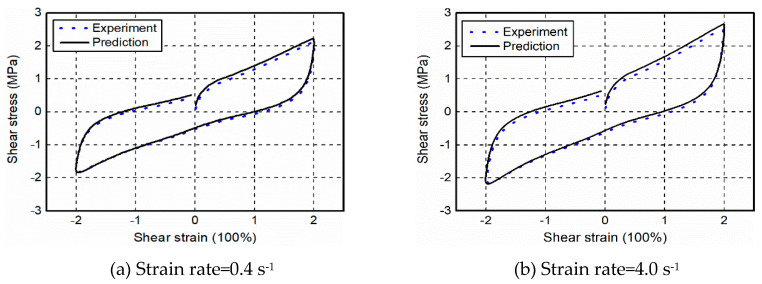
Comparison of cyclic shear tests and model predictions.

Figure 13 shows the comparison of experimental results and model predictions for the cyclic shear tests with two levels of strain rates, including 0.4 and 4.0 s^−1^—coherent agreement in terms of the stress-strain responses of the material between the experimental and numerical results is observed in both cases, indicating the favorable capacity and reliability of the proposed model in predicting the rate-dependent and cyclic behavior of filled rubber. Notably, the proposed model presents an error in small deformations. For instance, the stress predicted by the model was approximately 7% greater than that of experimental results at appropriately 40% shear strain in the case of strain rate = 4.0 s^−1^. It should be noted that the error merely exists in local regions and is not enlarged after the initial deformation. The reasons for this discrepancy are twofold: one is that the strain energy function of model yields a less satisfactory agreement with the strong nonlinear behavior of the material at small strains; the other is the appropriation of the viscosity coefficient.

Considering the hysteretic behavior (i.e., energy dissipation capacity) of the filled rubber plays a crucial role in civil engineering, the hysteretic area (unit of the area is ignored for simplicity) of stress-strain curves of the specimens obtained from Figure 13 was calculated and is given in Table 3 for a further comparison. It is found that the predicted hysteretic area is in good agreement with the experimental results, the relative error for both cases is within 5%. Overall, the agreement is sufficiently to substantiate that the proposed model is capable of yielding a reasonable description in engineering practices when a filled rubber-based structural device is incorporated.

## 6. Conclusions

This paper presented a micro-mechanism-based hyper-viscoelastic constitutive model to describe the behavior of filled rubber under large shear deformation and various velocity impacts. Parameters in the model were identified by conducting experimental tests and the accuracy of the model was verified by comparing the experimental and numerical results. Major conclusions can be summarized as below:

1. The constitutive model comprises two parts: the first captures the equilibrium and instantaneous responses of filled rubber; the second incorporates the decomposition of the deformation gradient tensor to correlate the overstress tensor with strain rate. The proposed model covers a wide range of conditions, including small to large shear deformations as well as low to high velocity impacts that the filled rubber is expected to undergo in engineering practices.
2. Considering the similar nature (i.e., nonlinear hyperelasticity) of equilibrium and instantaneous responses of filled rubber, a newly-developed polynomial strain energy function is applied for equilibrium and intermediate springs in the constitutive model. The proposed strain energy function has a relatively simple mathematical formulation and its parameters are related to the physical description of the material molecular network.
3. A “Gau-Poly” function isproposed to capture the nonlinear viscosity coefficient in the constitutive model, three-dimensional plot of the experimental and fitted results of the viscosity coefficientshows that the “Gau-Poly” function has a good generalization ability to predict the variation in viscosity coefficient with extensive ranges of strains and strain rates.
4. The accuracy of the proposed constitutive model was verified by comparing the experimental tests and numerical simulation. A reasonable agreement between the experimental and numerical results substantiated the validity of the proposed model. The model not only fills a theoretical gap by developing an advanced model of filled rubber, but also supplies an appropriate choice to engineers to describe the behavior of filled rubber under the considered conditions. In the future, it is expected that the model can be implemented in finite element codes as a novel user-defined model to facilitate the numerical simulation when designing structural devices that incorporate such materials.

## Figures and Tables

**Table 1:** Parameters of equilibrium stress and overstress (unit: MPa).

Stress Tensor	*C*_1_	*C*_2_	*C*_3_	*C*_4_	*C*_5_
Equilibrium stress	0.899	−1.146	0.678	−0.127	0
Overstress	2.484	−5.623	6.263	−3.075	0.548

**Table 2:** Parameters of viscosity coefficient.

*z*_0_ (MPa·s)	*A* (MPa·s)	*x_c_*	*w*_1_	*y_c_* (s^−1^)	*w*_2_ (s^−1^)	*b*	*c* (s)	*d* (s^2^)
0.346	1.559	1.090	0.551	6.607	6.030	4.194	3.779	0.052

**Table 3:** Hysteretic area obtained from experimental results and model predictions.

Case	Experimental Results	Model Predictions	Relative Error
Strain rate = 0.4 s^−1^	4.65	4.83	+3.87%
Strain rate = 4.0 s^−1^	5.46	5.73	+4.94%
